# Disconnection between sugars reduction and calorie reduction in baked goods and breakfast cereals with sugars-related nutrient content claims in the Canadian marketplace

**DOI:** 10.3389/fnut.2025.1539695

**Published:** 2025-02-24

**Authors:** Ye Flora Wang, Sandra Marsden, Chiara DiAngelo, Abigail Clarke, Anita Chung, Jessica Yu, Zhongqi Fan, Julian Cooper, David Kitts

**Affiliations:** ^1^Nutrition Information Centre, Canadian Sugar Institute, Toronto, ON, Canada; ^2^Department of Applied Human Nutrition, Mount Saint Vincent University, Halifax, NS, Canada; ^3^Dalla Lana School of Public Health, University of Toronto, Toronto, ON, Canada; ^4^Friedman School of Nutrition, Tufts University, Boston, MA, United States; ^5^342 Consulting Ltd, Dereham, Norfolk, United Kingdom; ^6^Faculty of Land and Food Systems, University of British Columbia, Vancouver, BC, Canada

**Keywords:** reformulation, sugar reduction, breakfast cereals, calories, baked goods, sugar claims

## Abstract

**Introduction:**

Nutrition claims aim to highlight key attributes in foods and assist consumers to make informed dietary choices. Consumers generally perceive products with claims related to lower sugars content as being healthier. Food manufacturers also use these claims to highlight reformulation action in response to consumer demands and government policies.

**Methods:**

A cross-sectional analysis of baked goods and breakfast cereals in the Canadian marketplace was conducted, focusing on the use of sugars-related nutrient content claims (i.e., “no added sugars,” “lower/reduced in sugars,” “sugar-free”) and changes in nutrients and energy content in reformulation strategies. Baked goods and breakfast cereals with sugars-related claims in Canada as of December 2022 were obtained from the Mintel Global New Products Database. Current product availability was verified using websites from manufacturers and major food retailers. Corresponding reference products were identified based on claim criteria specified by the Canadian Food Inspection Agency. Differences in energy, macronutrient content and key ingredients involved in sugars reformulation were assessed between claim and reference products.

**Results:**

A total of 111 baked goods and 23 breakfast cereal products were included. No significant difference was found in mean energy content between the claim and reference products for all subcategories, except for “unsweetened” baked goods, where the energy content in claim products was significantly higher than that of the reference products (*p* < 0.001). Specifically, 49% of products with claims of “no added sugar,” 27% of “sugar-free,” and 23% of “lower/reduced in sugar” had higher energy content compared to corresponding reference products. Sugar alcohols, dietary fibers, non-nutritive sweeteners and starch were the top ingredients used in place of added sugars in claim products.

**Conclusion:**

No significant difference in mean total energy content (per 100 g) between baked goods and breakfast cereals carrying sugars-related claims was found, despite various sugar reduction strategies. Thus, these claims could be misleading to consumers who expect such products to be lower in total calories. Food manufacturers are encouraged to reformulate products with improved calorie and nutrition profiles rather than using a single-nutrient focus. Consumers education on these issues can help them be mindful of the presence and unintended consequences of common sugar-replacement practices.

## 1 Introduction

Global concerns regarding the prevalence of obesity have sparked an increased interest in understanding potential associations with specific dietary components, among which sugars are a primary focus of research and media attention. Government policies such as the adoption of front-of-package labeling schemes in many countries including Canada, and the United Kingdom “Sugar Reduction Program” are encouraging manufacturers to reformulate existing products to provide more selections of foods lower in sugars to help reduce the risk of obesity and associated chronic diseases. Manufacturers often highlight reformulation efforts using sugars-related nutrient content claims such as “reduced in sugars” and “no added sugars.” Consumers also generally perceive products with nutrient content claims related to lower sugars content as a key attribute to define a healthy food and diet (1–3).

Excess calorie consumption beyond body requirement is a well-documented key risk factor for an increased risk of obesity and many metabolic disorders such as heart disease and type-2 diabetes. The association of dietary sugars with these metabolic health outcomes, as shown in systematic reviews and meta-analyses, suggests that energy intake plays a fundamental role in mediating the health impacts of sugars (4–7). Specifically, when reduced sugars intake resulted in lower total energy intake, improvements in body weight and associated disease risk factors were observed; but when energy from sugars was replaced with equivalent calories from other macronutrients, no difference was observed in major metabolic outcomes. Hence, reducing sugars content in foods without a corresponding reduction in calories will unlikely achieve the intended public health benefits of sugar reduction policies.

Previously in marketplace research comparing changes in sugars reformulated products between 2013 and 2017 using the Canadian Food Labelling Information Program (FLIP) database showed a lack of overall calorie reduction in products reformulated to be lower in sugars (8). A scan of the packaged food supply also showed that the association between the content of sugars and calories was not linear, but rather varied by food or beverage category and was dependent on the complexity of sugar’s functionality in the correpsonding formulation (8, 9). With continuing public health policies that encourage manufacturers to reformulate products to develop a healthier product composition, including reduced sugars, a careful review of the latest packaged food supply is needed to better understand sugars reformulation processes and associated changes in ingredients, energy and nutrient composition.

Grain products are the top energy provider for both children and adults in Canada (10). Baked goods and breakfast cereals are two major sources of grains in the diet and are also foods where sugars play versatile functional roles, creating technical challenges to achieve sugars reduction (11, 12). As a result, these are important food categories to assess sugars reformulation trends and strategies. This cross-sectional analysis of baked goods and breakfast cereals in the Canadian marketplace was focused on sugars-related nutrient content claims with respect to changes in energy and nutrient composition, and the list of ingredients, to better understand reformulation outcomes and to track market trends in the future.

## 2 Materials and methods

### 2.1 Data source

The Mintel Global New Product Database (GNPD) is a proprietary online database that tracks new product launches, new formulations and packaging (including foods and beverages) around the world with data dating back to 1996. It provides detailed package information, nutrition labeling, nutrition claims, and ingredients. Search criteria were applied to the Mintel database to identify breakfast cereals and baked goods carrying sugars-related claims that were available in the Canadian marketplace between January 2013 and December 2022. The sugars-related claims were organized by Mintel into the following categories: “low/reduced sugar,” “no added sugar,” and “sugar free.”

### 2.2 Product availability verification

The availability of Mintel identified claim products in the Canadian marketplace as of year-end 2022 was verified by extracting product information posted to manufacturer and/or retailer websites, public online forums, social media, and physical product packaging as required. All identified products existing in the Canadian marketplace as of year-end 2022 were marked as “verified.” If a product was not available at this cut-off point, the product was marked as “retired” and was excluded from further analysis. The resulting database is a cross-sectional snapshot of all baked goods and breakfast cereals that carry sugars-related claims in the Canadian marketplace.

### 2.3 Validation of sugar claims

Because Mintel categorizes sugars claims based on product packages, the validity of these claims needed to be verified against corresponding Canadian regulations and guidelines (i.e., Canadian Food Inspection Agency criteria for sugars-related nutrient content claims), in reference to an appropriate reference product not carrying the claim (13). Sugar claims of products included in this analysis were validated against the specific nutrient content claim requirements outlined in the Food and Drug Regulations (FDR) table following section B.01.513 of the 2022 version of the FDR, as these requirements will remain in effect until the amended nutrient content claim criteria come into force on 1 January 2026. As such, it was deemed appropriate to validate the claims carried on food products available as of December 2022 against the criteria that were in force at that time. Any discrepancies between the sugars claim(s) listed in the GNPD for a particular product and its packaging were reviewed by at least two co-authors to reach consensus. Products with valid claims were then categorized by claim category. Products carrying both “no added sugars” and “unsweetened” claims were categorized as the latter, because all products claiming to be unsweetened must meet the criteria for “no added sugars” as well as no other added sweetener. This included non-nutritive sweeteners and sugar alcohols.

### 2.4 Reference Product Identification

**FIGURE 1:**
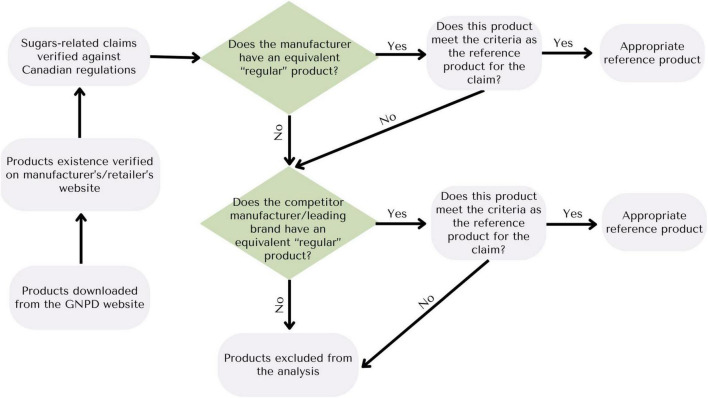
Decision tree for the identification of reference products.

For each verified claim product, reference products were identified to enable claim validation and comparative analyses (Figure 1). If a manufacturer did not explicitly identify a product for comparison, a similar reference product offered by the largest leading manufacturer/brand was selected. Verified claim products with no suitable reference product or with incorrect sugar claims were excluded from comparative analyses. New products and/or new flavors of existing products introduced to the Canadian marketplace within the specified period that were not listed in the GNPD were also collected during this process from manufacturers’ websites. These products were marked as “new products” and were included in analyses if inclusion criteria were met. Nutrition information from the Nutrition Facts table and the List of Ingredients of the selected reference products was recorded manually.

### 2.5 Statistical analysis

The differences in energy and macronutrient composition were determined by subtracting the level (per 100 g) of macronutrients in the claim product from the level of the same macronutrients in the reference product. Items with changes in sugars levels were further subdivided into those that had an increase, or a decrease in sugars. Descriptive statistics were used to compare changes in nutritional composition (i.e., energy, total fat, sugars, carbohydrate, starch, fiber, and protein) in grams per 100 g. Differences in the List of Ingredients were compared to identify plausible replacement ingredients for sugars for their functional roles. All analyses were conducted overall as well as stratified by claim products. The energy content between claim products and reference products within each type of claim was compared using Mann-Whitney U test. *P*-values < 0.05 were used to denote statistical significance. Analyses were conducted using R Statistical Software [v4.3.3; (14)].

## 3 Results

### 3.1 Differences in energy and macronutrient content between claim products and reference products

Baked goods and breakfast cereals with claims included in our analysis are summarized in Table 1. The differences in macronutrient and energy composition per 100 g between claim products and reference products are shown in Table 2. There was no difference in the energy content between claim and reference products for all subcategories. An exception was for “unsweetened” baked goods where the energy content in claim products was significantly higher than that of the reference products (*p* < 0.001). The average reductions in sugars ranged from 9.9 g to 38.1 g per 100 g in baked goods, which corresponds to a range of changes equitable to 40 kcal–152 kcal per 100 g from sugars (e.g., 4 kcal energy/gm sugar). However, the mean reduction in actual energy content was much less pronounced compared to the expected (with the exception of “reduced/lower in sugar” baked goods) (Table 2). A similar trend was observed for breakfast cereals, where the energy content in claim products was not significantly different from those of reference products. This can largely be explained by the increase in other food components such as the average content of dietary fibers, protein, fat and sometimes starch per 100 g, compared to the reference products.

**TABLE 1:** Categories of baked goods and breakfast cereals with sugars-related claims.

Category	Sub-category	No added sugars	Sugar-free	Unsweetened	Lower / reduced in sugars
Baked goods	Bread and Bread Products	22	4	0	0
	Sweet biscuits/cookies	15	2	0	4
	Baking ingredients and mixes	11	2	26	0
	Savory biscuits/crackers	8	4	0	4
	Cakes, pastries and sweet goods	1	7	1	0
Breakfast cereals	Cold cereals	10	2	0	5
	Hot cereals	5	1	0	0
Total		72	22	27	13

**TABLE 2:** Differences in macronutrient and energy composition between claim products and reference products.

	Difference in sugars g/100 g	Difference in energy from added sugars^2^ kcal/100 g	Difference in total energy kcal/100 g	Difference in carbohydrate g/100 g	Difference in fiber g/100 g	Difference in starch g/100 g	Difference in protein g/100 g	Difference in fat g/100 g	*P*^1^ for differences in energy content
**Baked goods**									
No added sugars (*n* = 57)	-14.4 ± 15.4	-57.4 ± 64.7	-7.2 ± 75.9	-10.1 ± 6.5	6.0 ± 15.9	10.9 ± 15.0	6.7 ± 7.9	3.8 ± 10.5	0.718
Unsweetened (*n* = 27)	-38.1 ± 11.8	-152.3 ± 47.4	150.2 ± 111.2	-25.3 ± 11.3	6.5 ± 6.0	6.5 ± 12.3	2.8 ± 2.4	28.3 ± 12.8	< 0.001
Sugar-free (*n* = 19)	-25.5 ± 21.1	-19.9 ± 4.4	-9.5 ± 90.4	-22.1 ± 13.4	10.4 ± 9.6	-7.0 ± 23.1	4.7 ± 7.2	11.9 ± 15.7	0.596
Lower/reduced in sugars (*n* = 8)	-9.9 ± 5.8	-39.7 ± 23.1	-40.8 ± 106.7	-8.4 ± 12.3	7.6 ± 6.5	27.2 ± 16.6	5.0 ± 4.8	1.0 ± 8.0	0.497
Total baked goods	-21.7 ± 18.3	-86.8 ± 73.2	28.3 ± 113.2	-15.7 ± 15.6	5.2 ± 7.5	3.3 ± 17.9	3.8 ± 6.6	10.5 ± 15.8	–
**Breakfast cereals**									
No added sugars (*n* = 12)	-11.8 ± 9.5	-47.2 ± 38.0	7.9 ± 27.0	-5.3 ± 6.3	2.7 ± 6.3	3.7 ± 10.7	2.2 ± 4.5	0.7 ± 1.6	0.197
Sugar-free (*n* = 3)	-20.0 ± 1.3	-80.0 ± 5.2	-21.6 ± 23.5	-3.9 ± 2.2	3.8 ± 0.2	12.2 ± 1.2	3.5 ± 2.1	0.4 ± 0.2	–^3^
Lower/reduced in sugars (*n* = 5)	-7.8 ± 5.2	-31.2 ± 21.8	-9.5 ± 9.5	-8.1 ± 7.4	1.2 ± 0.9	-1.5 ± 7.0	1.6 ± 1.3	2.6 ± 2.3	0.465
Total breakfast cereals	-11.5 ± 8.9	-46.0 ± 35.6	0.9 ± 24.0	-4.8 ± 5.9	2.0 ± 4.8	4.6 ± 9.8	2.0 ± 3.4	0.9 ± 1.8	–

Values are expressed as Mean ± Standard deviation.

^1^*P* denotes the level of significance in the difference in the energy content between claim products and the reference products for each claim category. Within each claim category, *p* < 0.05 if the energy content in the claim products is significantly different from that in the reference products.

^2^Calculated based on 1 g of sugars providing 4 kcal energy.

^3^Unable to calculate *p*-value due to the small sample size (*n* = 3).

### 3.2 Reformulation strategies by key ingredients in baked goods and breakfast cereals

Common substitution ingredients used in sugars-claim products are summarized in Table 3 by key functional roles in the product. The most frequently used ingredients were sugar alcohols, dietary fibers, non-nutritive sweeteners, and starch-based ingredients.

**TABLE 3:** Common sugar substitution ingredients used in products with different sugars-related claims based on functional roles.

Ingredient category	Key functional roles of sugars	Common examples in these categories	Sugars-related claims
Non-nutritive Sweeteners	Sweetness	Sucralose, aspartame, steviol glycosides	Sugar-free, lower/reduced in sugars
Starch	Texture, structure, moisture retention, gel formation	Wheat starch, corn starch, dextrin, glucose syrup, potato flour, maltodextrin	No added sugars, unsweetened
Fiber	Bulking, texture, structure, emulsifier, stabilizer, thickener	Inulin, guar gum, bran, polydextrose	Sugar-free, lower/reduced in sugars
Sugar alcohols	Sweetness, bulking, texture, structure, thickener	Erythritol, maltitol	Sugar-free, lower/reduced in sugars, no added sugars

### 3.3 Key ingredient changes in claim products with no energy reduction

**FIGURE 2:**
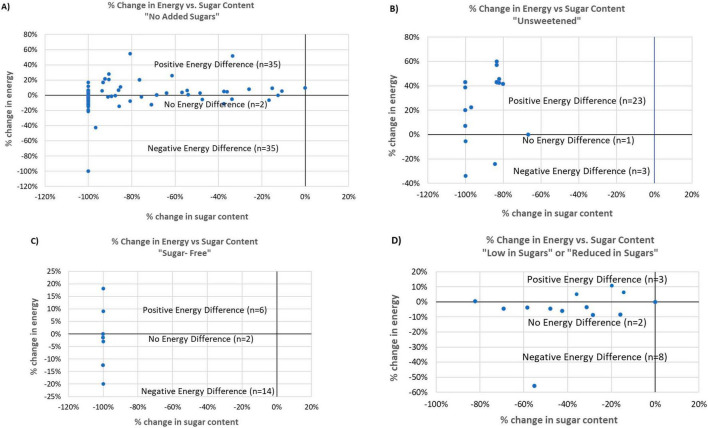
Scatter plots of % change in energy (E) against % change in sugars in four claim categories among baked goods and breakfast cereals. **(A)** No added sugars, **(B)** Unsweetened, **(C)** Sugar-free, **(D)** Low or reduced in sugars.

Among baked goods and breakfast cereals, higher calorie contents compared to corresponding reference products were found in 23 out of 27 (85%) products with “unsweetened” claims; 35 out of 72 (49%) products with “no added sugars” claims; 6 out of 22 (27%) products with “sugar-free” and 3 out of 13 (23%) products with “lower in sugars” or “reduced in sugars” (Figure 2). The most frequently used ingredient substitutions in claim products with higher calorie content were sugar alcohols (e.g., erythritol, maltitol), followed by starch-based ingredients (e.g., potato flour, wheat starch) (Table 3). For products with “no added sugars” claims, those with greater energy content generally had added starch, sugar alcohols, oils, or protein isolates as substitute ingredients compared to the reference product. Most “unsweetened” products with a greater energy content compared to the corresponding reference product, were baking ingredients such as unsweetened coconut and chocolate chips. The higher average energy content was due to a higher proportion of shredded coconut or cocoa butter, which have a higher energy density. The “sugar-free” claim products with higher energy content also had higher fat content, which contributed to the energy difference. In addition, some claim products that had a higher energy content compared to the reference product had added ingredients such as coconut oil and oil seeds that contributed to greater energy density compared to sugars.

## 4 Discussion

Global trends in product reformulation aimed to reduce sugars consumption are influenced by many factors, encompassing government policies, public health promotions, consumer demands and manufacturer innovations. To our knowledge, the present study is the first to assess sugars-specific reformulation efforts focusing on information related to replacement ingredients that can change both sugars and calorie composition in products using sugars-related nutrient content claims. Our study showed that in the majority of reformulated baked goods and breakfast cereals, the extent of calorie reduction was less than expected based on the reduction seen in total sugars levels. These observations are consistent with previous findings derived from a different Canadian product database (9). Thus, both studies reflect sugars-related product trends in the Canadian marketplace and the results are consistent with the observed limitations of sugar reformulation strategies in achieving energy reduction. For example, in the Public Health England Sugar Reduction-Industry Progress Report 2015–2020, the extent of sales-weighted average calorie reduction was shown to be markedly less than the extent of sugars reduction for each category reported (15).

The lack of a direct linkage between sugars reduction and calorie reduction in reformulated baked goods and breakfast cereals may be partially explained by the challenges in using substitute ingredients to replace the multiple functional roles sugars play in these food categories that go beyond sweetness, such as texture, mouthfeel, and control of water activity (12). Thus, product reformulation is generally a more complex process in foods compared to sugars-sweetened beverages, where the primary function of sugar is to provide sweetness and balance flavors. Alternative sweeteners (e.g., sucralose, aspartame, steviol glycosides) have been used extensively, alone or in combination, in beverages to replace the sweetness of sugar with little to no added calories. However, in foods, additional ingredients are often needed to replace the multiple functional roles of sugar while not compromising the sensory qualities considered acceptable to consumers. Beyond sweet taste, our results indicate that ingredients such as maltodextrin, modified starch, fats and oils, dietary fiber, and sugar alcohols are often added to maintain texture and provide bulk in cereals and baked goods. When multiple substitution ingredients are used, such as starches or fat, which have the same or higher caloric density compared to sugars, the overall goal of reducing calories by reducing sugars in the recipe can be diminished. In other food categories not examined in this study, there also exist products marketed to be lower in sugars without having a reduction in energy. For example, a popular brand of “less sugar” chocolate syrup, has the same amount of calories per serving compared to the regular chocolate syrup. The amount of carbohydrate is the same between these two products despite of the different amounts of sugars used. Analysis of the ingredients in these products shows that a proportion of sugar in the regular syrup is replaced with maltodextrin, a starch-based ingredient with an equivalent energy density to sugars, thus resulting in no change in total energy in the reformulated syrup. In many baked goods such as cookies where sugars and fats are used, a reduction of sugars in the recipes directly leads to a lower product weight and a greater proportion of energy contribution from fat. This results in a greater energy density of the “lower sugar” product and hence higher calories when compared on a same weight basis in relation to the original recipe.

In this study, 5 out of 13 (38%) baked goods and breakfast cereal products carrying the claim “reduced in sugar” and 37 out of 72 (51%) of those with “no added sugars” had no calorie reductions or an even higher calorie content compared to the corresponding reference products. When cumulative evidence consistently shows that the health impacts of dietary sugars are mediated by energy intake, reducing sugars without an equivalent reduction in calorie content may not achieve the intended public health benefits of these policies. To mitigate such a risk, better labeling regulations and guidelines may prove to be an effective tool. For example, in the European Union, the claim “reduced sugars” can only be made if the energy content is equal to or less than the equivalent similar product (16). In the United States, the statement “not a low calorie food” or “not a reduced calorie food” is required to accompany “sugar-free” or “no added sugars” claims if the products don’t meet the “low calorie” or “calorie reduced” claim criteria (17). This approach may provide a meaningful tool to direct consumers’ attention to the Nutrition Facts table for further information on sugars and calorie content.

Consumer eating behavior and food choices associated with sugar-reduced products have also been explored. A randomized cross-over dietary intervention showed that while consumers of sugar-reduced foods and beverages over an 8 weeks period had lower intakes of carbohydrate and total sugars, total energy intake was comparable to those who consumed regular-sugar foods and beverages. This observation was attributed to energy compensation (e.g., increased energy intakes from fat and protein) (18). Consequently, there was also no difference in body weight, blood pressure, fasting levels of glucose and lipids between both groups. From time to time, consumers underestimate both the energy and sugars content of foods and beverages in addition to non-intentional energy compensation (19–21). For example, “low-fat” claims have also been shown to contribute to underestimating the energy content of food products (22), which would be similar to the research highlighting consumer confusion regarding the level of calorie reduction associated with reduced sugar claims (3). This consideration further emphasizes the importance of consumer education in reading nutrition labels to fully understand the nutritional composition of different products, including calorie content, to avoid unintentional compensatory eating behavior (23). Practicing mindful eating and recognizing the importance of portion-size control when consuming foods such as baked goods and breakfast cereals are also important strategies as distinct from a singular focus on sugars or other nutrients to achieve energy and nutrient intake goals.

Beyond the calorie perspective, other important factors should not be overlooked when considering sugar reduction and product reformulation with other ingredients. Individuals with Type 2 Diabetes are generally recommended to adopt a low-glycemic index (GI) dietary pattern and limit the consumption of free sugars to less than 10% of daily energy intake (24). GI measures the amount of a carbohydrate-containing food, or drink, impacting changes in blood glucose levels after it is consumed and digested. A key contributor to the GI value is the amount of digestible carbohydrate in the food or drink, especially the glucose content. For example, starch-based ingredients are digested only into glucose, thus producing a relatively higher GI than many types of sugars that are composed of other monosaccharides such as fructose or galactose, in addition to glucose in various proportions. Based on our observation, the use of different types of starch-based ingredients in “low-sugar” products that are designed to make up for bulk, texture and food structure, result in not only narrowing the calorie gap initially created by the reduction of sugars, but also can unintentionally change the glycemic index of the final product. Further research and validation are warranted to better understand how different sugar-replacement ingredients can impact glycemic responses and overall product quality to better guide meaningful sugar reformulation strategies.

The use of sugar alcohols is prevalent in sugar-reduced baked goods, given the important role to contribute to changes in sweetness, bulk, and texture with lower calorie density compared to sugars. In this study, sugar alcohols were present in approximately 30% of “no added sugars” products and 50% of “sugar-free” products in this study, with an average of 5 and 4 g per serving, respectively. Many products listed sugar alcohols within the top three ingredients in the List of Ingredients information. While sugar alcohols are not absorbed in the small intestine, which underlies a comparatively low-calorie low-glycemic contribution when replacing sugars, the consequence of eating excessive sugar alcohols can lead to gastro-intestinal discomfort and laxative effects in adults, given poor absorption in the gastrointestinal tract. The tolerance level in children may be even lower. While there is a wide variation in sensitivity between individuals to these effects, the likelihood of these events occurring is related to the amount consumed and, therefore, could increase with the consumption of more than one product containing sugar alcohols. Currently in Canada, regulations only require the quantity (grams per serving) of sugar alcohols to be declared in the Nutrition Facts table under “Total Carbohydrate,” but without any warning in case of an increased risk for high consumption (25). In the United States, the declaration of sugar alcohols on the Nutrition Facts table is voluntary, but it becomes mandatory when the packaging makes claims about sugars or sugar alcohols (e.g., “sugar-free”) (26). The European Union takes a further step to require that foods containing any sugar alcohols (polyols) carry the statement “with sweetener(s)” and with the quantity given in the nutrition declaration. If there are more than 10% sugar alcohols, a warning label of “excessive consumption may produce laxative effects” must be clearly displayed (16). It is therefore important to ensure informative labeling practices to be used, including the consideration of front-of-package disclaimers, as seen in the EU systems, to properly inform consumers of potential side effects of sugar alternatives (27). Another noteworthy observation in this study was that there was an average increase in the amount of soluble, non-digestible dietary fiber in claim products; which was attributed to the use of ingredients such as inulin and polydextrose. These specific ingredients provide similar bulk and moisture retention properties to maintain the structure and mouthfeel of the product when sugar is reduced (28). Moreover, they are also regarded as prebiotics with a function to promote a healthy gut microbiome, in addition to potentially reducing the glycemic index of the claim product, by slowing the digestion and reducing absorption of carbohydrate in the small intestine (28). Consumers who may be sensitive to a sudden introduction of large amounts of fermentable fiber-based ingredients, may experience gastrointestinal side effects, such as bloating or diarrhea. Better consumer tolerance testing could be considered as part of thoughtful product development by manufacturers (29).

Lastly, food sources of sugars should also be considered as part of sugar reformulation efforts. Sugars-sweetened beverages, the top contributor of dietary sugars, are often linked to contribute to excess energy intake, or negative health outcomes (30–33). On the contrary, certain categories of sugars-sweetened foods such as cereal grain products contribute positively to the nutrient density and a healthy body weight outcome. Ready-to-eat cereals, for example were reported to contribute to improved nutrient intakes providing one-third of daily iron intake and at least 10% of dietary fiber, thiamin, folate and vitamin B_6_ in Canadian adults and children (34). A systematic review and meta-analysis further suggested that the consumption of ready-to-eat breakfast cereals in children and adolescents did not increase body weight or body composition, regardless of whether they were sweetened or unsweetened (35). Added sugars intakes from sweet bakery products among United States adults are positively associated with higher quality of the overall diet (36). These grain-based foods play a vital role in improving whole grain and concomitant dietary fiber and micronutrient intake, whereas the addition of sugars in the recipe could help improve the overall palatability of whole grain foods beyond sweetness by balancing sometimes bitter, or earthy flavors of whole grains, retaining moisture and extending shelf-life, and in appropriate cases facilitating proper yeast fermentation. Therefore, compared to a relatively straightforward approach to sugar reformulation in sugars-sweetened beverages, it is debatable if it is worthwhile to reformulate sugars in grain products in the absence of achieving a meaningful calorie reduction as well as other beneficial effects directed at food quality.

A key strength of this study is that the data presented are based on Mintel GNPD, which provides the most up-to-date Canadian marketplace data to track sugars reformulation efforts. It can serve as a baseline for tracking ongoing reformulation efforts in baked goods and cereal products following the introduction of Front of Package labeling regulations. Several limitations of the study should also be considered while interpreting our results. Firstly, Mintel GNPD does not remove retired products; therefore, manual verification was required to ensure that the products included in our analysis reflect current marketplace availability. Certain non-CFIA defined sugar claims such as “sugar-wise” were used in several ready-to-eat cereals, and “lightly sweetened” in an oatmeal product. As a result, we were unable to categorize them in our analysis due to unclear definitions of these manufacturer-defined claims. There may also be products not captured by the database. The sugars claim categorization by Mintel was based on United States definitions, which required further validation against CFIA criteria. Secondly, although every effort was made to identify a suitable reference product, certain claim products were compared to a reference product of a leading brand but from a different manufacturer. The differences in nutrition content between the claim and reference product may be the result of different recipes rather than product reformulation.

In conclusion, our study showed, that there was frequently a lack of energy reduction in baked goods and breakfast cereals bearing sugars related claims despite various strategies to reduce sugars content. We conclude that these claims potentially mislead consumers who expect such products to be lower in calories. Consumers should be encouraged to look at the entire food package, including the List of Ingredients, Nutrition Facts table, and nutrient content claims, rather than solely focusing on the sugars claim, in order to better understand the complete nutrition profile and to choose a product that meets needs and preferences. Food manufacturers should also carefully consider reformulation strategies and provide products with an improved calorie and nutrition profile rather than simply emphasize sugars reduction.

## Data Availability

The raw data supporting the conclusions of this article will be made available by the authors, without undue reservation.
